# Consortia formed by yeasts and acetic acid bacteria *Asaia* spp. in soft drinks

**DOI:** 10.1007/s10482-017-0959-7

**Published:** 2017-10-20

**Authors:** Dorota Kregiel, Steve A. James, Anna Rygala, Joanna Berlowska, Hubert Antolak, Ewelina Pawlikowska

**Affiliations:** 1 Institute of Fermentation Technology and Microbiology, Lodz University of Technology, ul. Wolczanska 171/173, 90-924 Lodz, Poland; 2 National Collection of Yeast Cultures, Quadram Institute Bioscience, Colney Lane, Norwich Research Park, Norwich, NR4 7UA UK

**Keywords:** Yeasts, *Asaia*, Hydrophobicity, Consortia, Soft drinks

## Abstract

Yeast strains and acetic acid bacteria were isolated from spoiled soft drinks with characteristic flocs as a visual defect. Polymerase chain reaction and amplification of a partial region of the LSU rRNA gene identified the bacteria as *Asaia* spp. Sequence analysis of the D1/D2 region of the 26S rDNA in turn identified the yeast isolates as *Wickerhamomyces anomalus*, *Dekkera bruxellensis* and *Rhodotorula mucilaginosa.* The hydrophobicity and adhesion properties of the yeasts were evaluated in various culture media, taking into account the availability of nutrients and the carbon sources. The highest hydrophobicity and best adhesion properties were exhibited by the *R. mucilaginosa* cells. Our results suggest that *Asaia* spp. bacterial cells were responsible for the formation of flocs, while the presence of yeast cells may help to strengthen the structure of co-aggregates.

## Introduction

Spoilage is a metabolic process that causes beverages to become either unfit for human consumption or unacceptable to consumers. The critical parameters concerning microbial spoilage are: pH, oxidation/reduction potential, water activity, the availability of nutrients, the presence of antimicrobial compounds and competing microbiota. Soft drinks support the growth of a limited number of microorganisms. They are contaminated mainly by acid-tolerant bacteria and fungi, which use the ingredients of the beverage as substrates for growth. Bacterial genera often reported include *Acetobacter*, *Gluconobacter* and other acetic acid bacteria (AAB). *Asaia* spp., increasingly isolated from commercial soft drinks, were recognized relatively recently as spoilage bacteria (Moore et al. 2002; Horsáková et al. 2009; Kregiel et al. 2012).

Yeast spoilage is common in soft drinks. High-acid, low-pH soft drinks with high sugar content, preserved using weak organic acids and bottled in plastic packaging are particularly prone to yeast spoilage. Yeasts belonging to the genera *Pichia, Candida, Saccharomyces* and *Rhodotorula* are commonly encountered as spoilage agents in soft beverages (Aneja et al. 2014; Kregiel 2015).

Our cooperation with Polish drinks factories shows that microbial growth often occurs despite the low pH of beverages and the addition of chemical preservatives (benzoate, sorbate or dimethyl decarbonate within legal limits) (Kregiel 2015). After 5–14 days of storage, spoiled soft drinks may become turbid (equivalent to McFarland standard ~ 3 or more) or contain characteristic ‘flocs’. These undesirable changes may be caused by a combination of yeasts and acidophilic bacteria. According to the literature, cell consortia formed by these taxonomically distinct groups of microorganisms are widespread in nature, in proportions that depend on a variety of environmental factors as well as on the surface characteristics of the microbial cells (Barata et al. 2014; Marsh et al. 2014). The stability of such microbial consortia depends *inter alia* on the adhesion abilities of the individual members of the consortium (Kreth and Herzberg 2015).

Controlling the growth and activity of spoilage microorganisms requires a good understanding of their ecology, their physiology and their ability to form specific consortia with other participating microbiota. Unfortunately, there remain large gaps in our knowledge of these areas, especially with regard to AAB species and yeasts other than *Saccharomyces cerevisiae*. In this study, we investigated the properties of yeasts isolated as co-cultures from spoiled soft drinks. The objective was to evaluate the hydrophobicity and biofouling tendencies of yeasts forming consortia with *Asaia* spp. cells in spoiled soft drinks.

## Materials and methods

### Microbiological analysis of soft drinks

In total, 25 samples were subjected to microbiological analysis: 15 samples of fruit-flavored mineral water packaged in polyethylene terephthalate (PET) bottles, and ten samples of drinks in glass bottles, sourced from different Polish manufacturers between 2011 and 2016. These commercial products were purchased on the market (without visual defects) or supplied by manufacturers for testing (with visual defects). All soft drinks were tested at least 2 weeks after bottling and storage at 20–25 °C. Qualitative examination of the 25 samples was conducted using the conventional pour plate method and GC agar [0.3% peptone (w/v), 0.3% yeast extract (w/v), 0.7% CaCO_3_ (w/v); 2% agar (w/v)] with d-glucose or sucrose 2% (w/v) (Kregiel et al. 2012). For each soft drink studied at least three plates were inoculated. Visually defective samples with flocs were plated directly by inoculation (0.1 mL), while visually clear samples were filtered (0.45 µm, 20 mL). All plates were incubated at 25 °C for 5 days. At least five characteristic colonies representing each morphotype obtained were picked up from the agar plates, re-streaked several times to ensure purity and then maintained as pure cultures at 4 °C on wort agar slants.

### Identification of microorganisms

Bacterial cultures were identified initially by light microscopy, and then using the following standard methods: Gram staining, the aminopeptidase test (Bactident Aminopeptidase, Merck), the oxidase test (Bactident Oxidase, Merck) and the catalase test (Bactident Catalase, Merck), as well as molecular methods. The 16S rRNA genes were amplified through PCR according to the method described in our previous study (Kręgiel et al. 2014). The nucleotide sequences were then compared with 16S rRNA gene sequences obtained from the National Center for Biotechnology Information (NCBI) using the program BLASTN 2.2.27 + (http://blast.ncbi.nlm.nih.gov/Blast.cgi). The sequences were deposited in the GenBank database and assigned accession numbers (Kregiel et al. 2012, 2014).

Yeast cultures were detected using standard light microscopy, and species identification was determined by large subunit (LSU) rRNA gene sequencing, conducted by the National Collection of Yeast Cultures (Norwich, United Kingdom). The variable D1 and D2 domains of the LSU rRNA gene were PCR-amplified directly from whole yeast cell suspensions following the procedure and PCR parameters as described previously by James et al. (1994). The yeast LSU D1/D2 domain was amplified and sequenced using the conserved fungal primers NL1 (GCATATCAATAAGCGGAGGAAAAG) and NL4 (GGTCCGTGTTTCAAGACGG) (O’Donnell 1993). The amplified LSU D1/D2 products were purified using a QIAGEN QIAquick PCR purification kit, according to the manufacturer’s instructions. Direct sequencing of the purified LSU D1/D2 PCR products was performed using a Taq DyeDeoxy terminator cycle sequencing kit (PE Biosystems) and an Omnigene thermal cycler (Hybaid), according to the manufacturer’s recommendations. Purified sequencing reaction mixtures were electrophoresed using a PE Biosystems model 373A automated DNA sequencer. The LSU D1/D2 sequence of each yeast strain was compared with sequences held in the EMBL/GenBank sequence databases.

### Yeast cultivation

The yeast isolates were stored in liquid wort medium (Merck). For shake cultures, conventional liquid media containing either glucose or sucrose as a carbon source and other organic compounds were prepared. The minimal medium M1 [2% glucose or 2% sucrose (w/v), 0.1% (NH_4_)_2_SO_4_ (w/v), 0.3% KH_2_PO_4_ (w/v), 0.2% MgSO_4_ × 7H_2_O (w/v), 0.05% yeast extract (w/v)] and enriched medium M2 [2% glucose or 2% sucrose (w/v), 0.1% (NH_4_)_2_SO_4_ (w/v), 0.3% KH_2_PO_4_ (w/v), 0.2% MgSO_4_ × 7H_2_O (w/v), 0. 5% yeast extract (w/v)] were sterilized at 121 °C, and the M3 medium [8.1% sucrose (w/v), 0.05% strawberry flavor (w/v), 0.16% citric acid (w/v), 0.02% sodium benzoate (w/v), 0.02% dimethyl decarbonate (velcorin) (w/v)] was sterilized by filtration using 0.45 μm-pore-size filters (Millipore). The culture medium (20 mL) was then poured into sterile 25 mL Erlenmeyer flasks, into which sterile glass carriers (microscope slides, Star Frost 76 × 26 mm, Knittel Glass, Germany) were placed vertically in such a way that half of the carrier was immersed in the medium, while the other part remained outside. The glass carrier was chosen as a reference hydrophilic surface, on which adhesion of *Asaia* spp. was weaker in comparison to the plastic carriers (Kregiel 2013a; Kregiel et al. 2014; Antolak et al. 2016).

The amount of inoculum was standardized to obtain a cell concentration in the culture medium approximately equal to 5000–10,000 CFU/mL at the start of each experiment. The samples were incubated at 25 °C on a laboratory shaker (135 rpm) for 6 days (Kregiel 2013a).

### Yeast cell adhesion

Analysis of the extent of cell adhesion to the glass carriers was performed using luminometry and microscopic observations. For the luminometric tests, the carrier was removed from the culture medium, rinsed with sterile distilled water and swabbed using free ATP sampling pens (Merck). The measurements were reported in relative light units (RLU) using a HY-LiTE 2 luminometer (Merck). In the microscopic studies, the yeast cells on each carrier were stained with basic fuchsin (0.5%) and observed using an OLYMPUS type BX41 light microscope connected to a DP72 digital camera. The total cell adhesion area in the observation field was evaluated using UTHSCA Image Tool software (http://compdent.uthscsa.edu/dig/itdesc.html) (Kregiel 2013a).

### Determination of hydrophobicity

The MATH test was used to determine the ability of yeast cells to adhere to hydrocarbons, as a measure of their hydrophobicity. Yeast cultures were harvested by centrifugation at 5000 rpm for 5 min at 4 °C, washed twice in PBS and finally re-suspended in the same buffer. The cell suspension was adjusted to an A_540 nm_ value of approximately 1.0 with PBS buffer, then 2 mL of the yeast suspension was added to 0.4 mL of xylene (Merck) and vortexed for 60 s. The two phases were allowed to separate for 5 min at 25 °C. The aqueous phase was carefully removed and the A_540 nm_ was measured. The decrease in the absorbance of the aqueous phase was taken as a measure of cell surface hydrophobicity (CSH), which was calculated using the formula CSH (%) = [(A_0_ − A)/A_0_] × 100, where A_0_ and A are the absorbance before and after extraction with xylene, respectively. In terms of their percentage affinity to xylene, each yeast isolate was classified on a scale where < 10% = hydrophilic, 10–29% = medium hydrophilicity, 30–54% = medium hydrophobicity to > 55% = highly hydrophobic (Kregiel 2013a).

### Statistical method

The mean results from three independent experiments were calculated. Comparisons between the mean values were performed using the one-way ANOVA test (STATISTICA 10, StatSoft, Poland) followed by Tukey’s multiple comparison test.

## Results and discussion

### Microbiota of flocs

In this study, we researched a representative group of soft drinks in Poland, to investigate the possible extent of contamination by *Asaia* spp. and yeasts in commercial products. Generalization is an essential component of the wider scientific process. Under ideal conditions, all commercial soft drinks would be included in the study. Of course, this is not feasible. However, bacteria belonging to the genus *Asaia* are increasingly detected in spoiled soft drinks, and they are not the only kind of spoilage microorganisms in non-alcoholic beverages (Kregiel 2015). As a result, manufacturers have taken stringent steps aimed at preventing the appearance of defective products on the market. The limited number of samples (25) used in our study is not, therefore, a result of rare contamination by acetic acid bacteria *Asaia* spp., but rather a reflection of the seriousness with which this issue is taken by manufacturers. Similar tests have been performed for companies interested in unknown spoilage microorganisms, which are difficult to detect and identify.

**Fig. 1:**
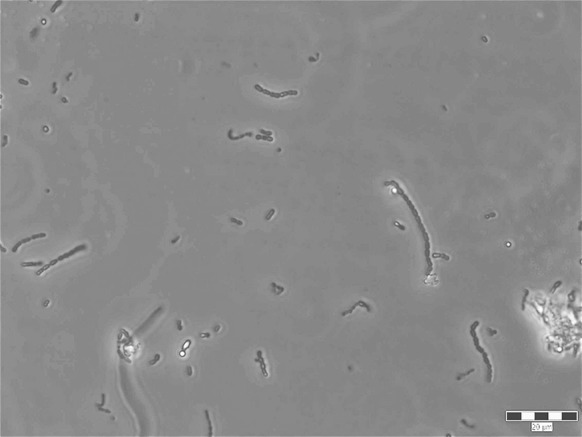
Microbiota of spoiled soft drinks. Flocs, free bacterial cells in chains and budding yeasts. Bar represents 20 μm

Only five samples of spoiled soft drinks packaged in PET bottles had characteristic, visually observable flocs, while others were visually clear or had slight turbidity (Table 1; Fig. 1). The levels of microbial cells in the contaminated samples were measured at 10^4^–10^6^ CFU/mL and 3–10^2^ CFU/mL, respectively. Yeast monocultures and AAB were isolated from three samples of the tested soft drinks packed in PET bottles, using the plate reduction technique on GC agar with glucose.

**Table 1:** Microbiota of flocs in soft drinks

No.	Type of soft drink	Visual defects	Number of morphotypes	Isolated AAB	Isolated yeasts
1	Fruit flavored mineral water	Flocs	2	*Asaia lannensis* HQ917852	*Wickerhamomyces anomalus* LT908480
2	Fruit flavored mineral water	Flocs	2	*Asaia lannensis* HQ917850	*Dekkera bruxellensis* LT908481
3	Fruit flavored mineral water	Flocs	1	*Asaia* sp. HQ917851	Not isolated
4	Fruit flavored mineral water	Without	1	*Asaia bogorensis* KT751285	Not isolated
5	Fruit flavored mineral water	Without	1	*Asaia bogorensis* KT751284	Not isolated
6	Isotonic drink	Turbidity	1	*Asaia bogorensis* KP234015	Not isolated
7	Isotonic drink	Turbidity	1	*Asaia bogorensis* KP7234014	Not isolated
8	Fruit flavored mineral water	Flocs	1	*Asaia lannensis* KP234012	Not isolated
9	Fruit flavored mineral water	Without	1	*Asaia lannensis* KP234011	Not isolated
10	Fruit flavored mineral water	Flocs	2	*Asaia bogorensis* KC756841	*Rhodotorula mucilaginosa* LT908482

**Fig. 2:**
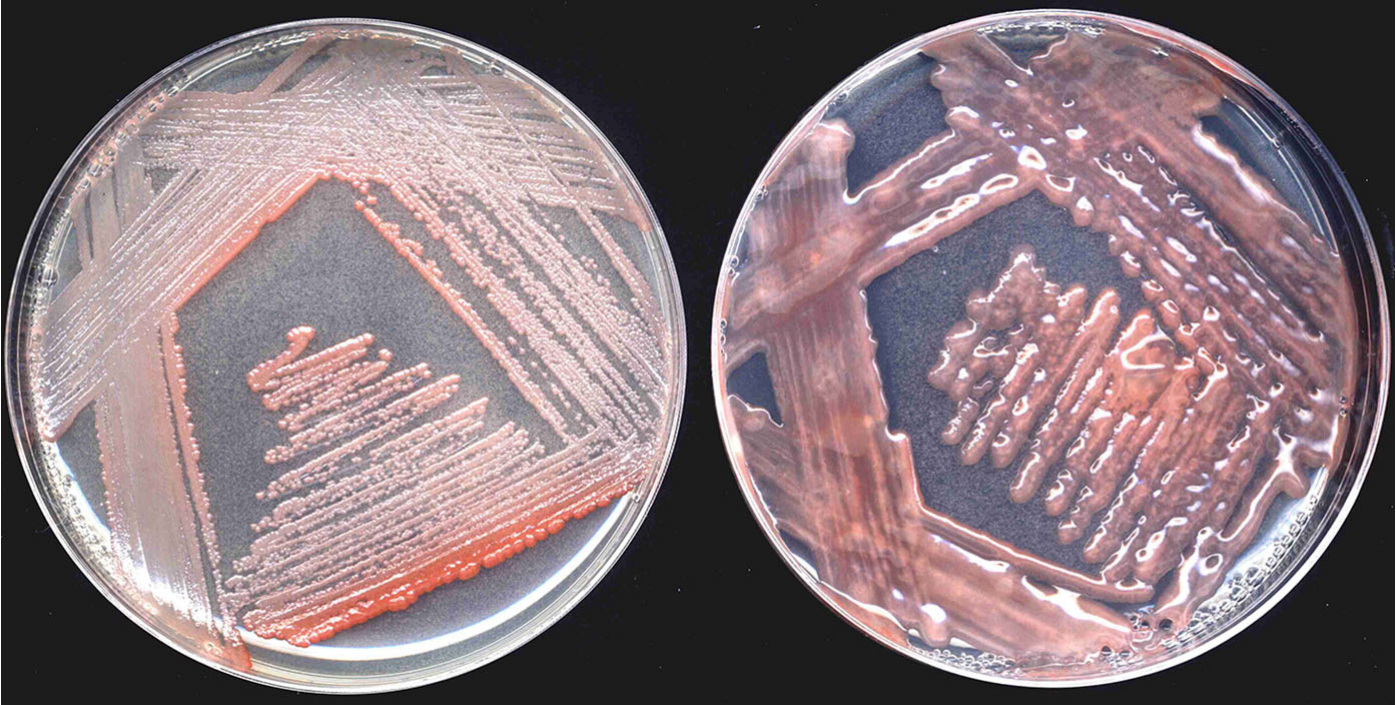
*Asaia* sp. colonies on GC agar with glucose (left) and sucrose (right)

**Fig. 3:**
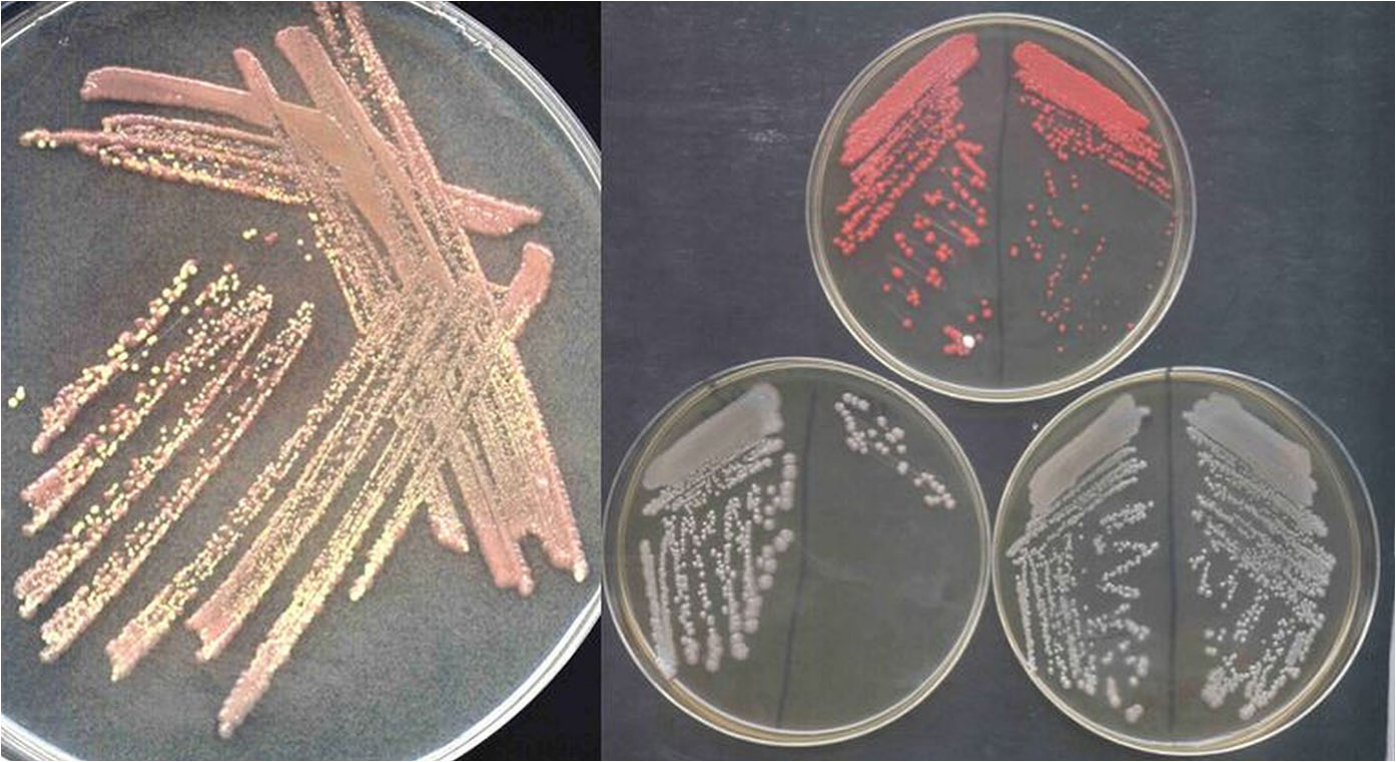
Streak plate method and three different yeast morphotypes after isolation

The bacterial morphotypes formed characteristic pink or pale pink to colorless colonies. These morphotypes were gram-negative, catalase positive, oxidase negative rods, identified as AAB belonging to the genus *Asaia*. However, three samples of the spoiled soft drinks with characteristic flocs were found to be mixed cultures of *Asaia lannensis* and accompanying yeasts. These formed light cream and red-colored colonies (Kregiel et al. 2012, 2014). Flocs were also formed by monocultures of *Asaia* spp. (*Asaia* sp. HQ917851, *A. lannensis* KP234012), but these structures were mechanically unstable and easy to damage by the normal intense vortexing. Mixed populations of *Asaia* spp. and yeasts were very difficult to separate from each other, especially using GC agar with sucrose as the culture medium. Isolated AAB on this agar formed numerous extracellular substances, and as a result observation of individual colonies was impossible (Fig. 2). The streak plate procedure was conducted several times on GC agar with glucose to ensure that the morphotype obtained was a monoculture (Fig. 3).

Each yeast monoculture was identified to species level by sequencing the D1/D2 domains of the large subunit rRNA gene. The isolated yeasts were identified as *Wickerhamomyces anomalus*, *Dekkera bruxellensis* (syn. *Brettanomyces bruxellensis*) and *Rhodotorula mucilaginosa*. In the case of the *D. bruxellensis* LT908481 isolate, this yeast displayed a notable level of sequence variation with the species type strain, suggesting it may belong to an as yet undescribed closely related sibling species. However, further research will need to be carried to investigate this possibility and to establish its taxonomic status.

Neither *Asaia* spp. nor yeasts were detected in soft drinks packaged in glass bottles. These products had been packaged using ‘hot filling’, unlike the ‘cold-bottled’ drinks in PET packaging. The method of bottling is known to be crucial for the level and type of spoilage microbiota in the final product. It is also important to note that the plastic packages were partially air-permeable (Kregiel 2015).

Yeasts and bacteria formed consortia in visually observable flocs. According to the literature, cell aggregates are usually formed by distinct microbial cells, with highly specific recognition and adhesion properties. The specificity of of coaggregating cells is mediated by complementary protein adhesins and polysaccharide receptors on the cell surfaces. This phenomenon is distinct from autoaggregation, which is the recognition and adhesion of genetically identical microorganisms (Vornhagen et al. 2013).

Microbial systems in nature are inherently complex and difficult to predict. Recent studies have revealed that yeasts and bacteria often form physically and metabolically interdependent consortia (Frey-Klett et al. 2011; Marsh et al. 2014; Gänzlea and Ripari 2016). An example of a system containing both AAB and yeasts is Kombucha, a traditional, fermented tea which originated in Asia. This drink is an excellent example of microbial ecology including consortia of yeasts and acetic acid bacteria. Our results confirm that in natural environments microbial consortia can be composed of one or more species. We observed previously unknown consortia in some soft drinks created by little studied AAB of the genus *Asaia* and yeasts.

The primary ecological niches of *Asaia* spp. have been reported as being in the flowers of the orchid tree (*Bauhinia purpurea*), in plumbago (*Plumbago auriculate*) and in fermented glutinous rice, all originating in hot tropical climates, particularly in Indonesia and Thailand (Kregiel et al. 2012). These bacteria have established symbiotic relationships with several insects which rely on sugar-based diets, such as nectars, fruit sugars or phloem sap (Crotti et al. 2009). It is difficult to say with certainty how *Asaia* spp. bacteria enter spoiled bottled soft drinks. However, given that they contain natural fruit juices, it may be speculated that the natural fruit juices are the probable source of this spoilage organism (Moore et al. 2002; Kregiel et al. 2012). The bacteria often form biofilms as well as cell aggregates, especially in the presence of sucrose. In our previous studies, the cell surfaces of *Asaia* spp. bacteria showed hydrophilic properties. However, the bacterial cells that formed flocs were found to be more hydrophobic than free cells (Kregiel 2013a). This may help them to co-aggregate with other hydrophobic microbial cells and strengthen the structures they form.

No less interesting is the presence of yeasts associated with *Asaia* spp. consortia in spoiled soft drinks. W. *anomalus* has been isolated from diverse plant habitats, and recently even from insects. It was detected in the midgut and gonads of *Anopheles stephensi* (Cappelli et al. 2014). This fact is particularly noteworthy, given that AAB belonging to the genus *Asaia* have been found in similar habitats (Favia et al. 2008). Therefore, the presence of *W. anomalus* and *A. lannensis* in flocs formed in fruit-flavored mineral water may indicate that these microorganisms share a common origin, although further research is required to explore this hypothesis.

It is also worth noting that the yeast *W. anomalus* is highly competitive in natural environments, and is known to control a range of post-harvest fungi, decreasing sporulation and mycotoxin production (Coda et al. 2011). Several strains of this species are able to produce killer toxins with broad activity spectra. Of particular interest are their anti-*Candida* and anti-mold activities (Farkas et al. 2012). *D. bruxellensis* (syn. *B. bruxellensis*) lives on the skins of fruits. This yeast is acidogenic, and when grown on glucose rich media under aerobic conditions can produce large amounts of acetic acid. It is considered a major worldwide cause of wine spoilage. Infected wines develop distinctive, unpleasant aromas due to the production of volatile phenols. Yet, the yeast is also known for its positive contribution of acetic acid flavor to both Belgian Lambic beers and the fermented tea Kombucha. Several strains have also been isolated from other sources, including apple cider and sweet drinks (Gamero et al. 2014). *Dekkera* (syn. *Brettanomyces)* spp. often accompany acetic acid bacteria (Schifferdecker et al. 2014). Their presence with *Asaia* spp. in isotonic drinks may be a result of their similar provenance from fruit, as well as of their presence inside the production line, especially where different drinks (including those containing alcohol) are produced, or where procedures for cleaning and disinfection are ineffective.

*Rhodotorula* species are easily identifiable by their distinctive cream, orange, red or pink colored colonies, and they are widely distributed throughout nature. *Rhodotorula* spp. have been isolated from various environmental sources, including soil, air, aquatic ecosystems, plants and fruits (Nunes et al. 2013). *R. mucilaginosa* is commonly isolated from foods and beverages. Several studies have reported the presence of *R. mucilaginosa* in apple cider, cherries, fresh fruits and fruit juices (Wirth and Goldani 2012). *Rhodotorula* spp. are considered as non-desirable yeasts by many winemakers. However, they are not necessarily considered spoilage yeasts, since they can be found in both many vineyards and winery environments. *R. mucilaginosa* has been reported as a saprobe from both skin and respiratory specimens, and its presence in soft drinks can signify cross-contamination during production. This yeast has a strong affinity for plastic materials, and spoilage of beverages may also occur via contaminated containers (Jimoh et al. 2012; Wirth and Goldani 2012).

In our study, yeast isolates formed stable consortia with *Asaia* acetic acid bacteria in soft drinks. It had been previously reported that hydrophilic cells of *Asaia* spp. exhibited a remarkable ability to co-aggregate/aggregate and form biofilms (Kregiel 2013a; Sedláčková et al. 2011). We therefore investigated these properties in yeasts isolated from consortia formed with *Asaia* spp. cells.

### Hydrophobicity of yeast cells

**Fig. 4:**
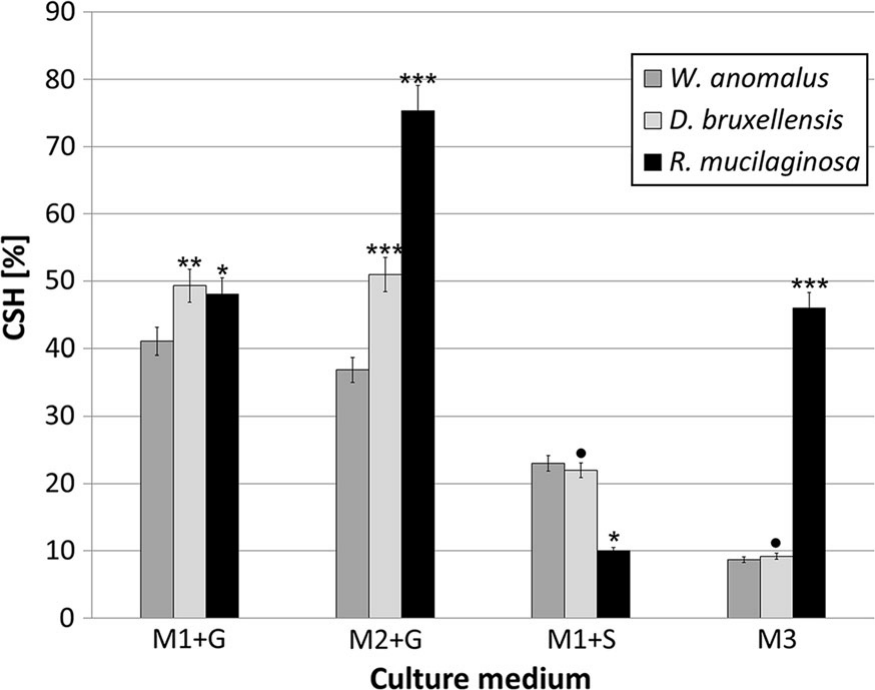
Cell surface hydrophobicity (CSH) of yeast isolates after 6-day incubation. Data analyzed with One-way ANOVA followed by Tukey’s test. ^•^p ≥ 0,05; *p < 0.05; **p < 0.01; ***p < 0.001 (data were compared to those received for *W. anomalus*)

Hydrophobic/hydrophilic interactions play an important role in both cell aggregation and adhesion to different surfaces. Unfortunately, most studies on yeast hydrophobicity have to date focused on flocculating *Saccharomyces cerevisiae* and pathogenic yeasts, especially of the genus *Candida* (Borghi et al. 2011). Cell surface hydrophobicity (CSH) is an important characteristic or trait, especially in terms of microbial ability to contaminate food products. It is one of the critical parameters conditioning adhesion to other microbial cells as well as to different abiotic surfaces. Some studies have suggested that measuring CSH could be a useful way to predict biofilm formation (Katsikogianni and Missirlis 2004). In our study, we therefore assessed the hydrophobic properties of yeasts isolated from different spoiled soft drinks (Fig. 4).

Our experiments on 6-day yeast populations confirmed that all the yeast strains were characterized by cell hydrophobicity. According to other studies, the degree of hydrophobicity may depend strongly on the chemical composition of the culture medium (Dengis et al. 1995). This fact was confirmed in our results. *R. mucilaginosa* displayed the strongest hydrophobic properties (p < 0.001), especially in commercial flavored water (M3) and enriched medium with glucose (M2 + G). It has been documented that *Rhodotorula* spp. are able to produce lipids and slime substances (Sampaio et al. 2001). These compounds may enable the yeast cells to attach themselves to both biotic and abiotic surfaces. The high hydrophobicity of *Rhodotorula* cells in M2 medium (with additional supplementation with yeast extract) may be connected to the accumulation of extracellular substances on the yeast cell surface. Gattlen et al. (2011) reported that biofilm formed by *R. mucilaginosa* cells over 6 days, isolated from washing machines, also contained proteins and polysaccharides.

The levels of CSH for *W. anomalus* and *D. bruxellensis* were comparable. A significant reduction in cell hydrophobicity was observed when the yeast cells were incubated in minimal medium with sucrose (M1 + S), although the lowest values were noted in commercial flavored water (M3). *Asaia* spp. are also able to moderate their CSH in culture media (Kregiel 2013a).

Microbial aggregation or adhesion to surfaces involves physico-chemical phenomena, which can effectively mask the influence of CSH as a biological factor. Glucose, sucrose and other sugars differ in terms of their lipo/hydrophilicity (Mazzobre et al. 2005). Therefore, sugars and yeast metabolites may influence CSH in culture media. Despite this fact, bacterial *Asaia* spp. form flocs in flavored mineral water, probably due to the formation of extracellular polymeric substances (EPS) (Kregiel 2013a). It can be assumed that EPS stimulate the formation of aggregates by bacteria and yeasts, and that the hydrophobicity of yeast cells strengthens the structures of the aggregates produced. In Kombucha tea, a characteristic dense ‘symbiotic colony’ is formed by AAB and yeasts belonging to *D. bruxellensis, Candida stellata, Schizosaccharomyces pombe, Zygosaccharomyces bailii* and other species (Teoh et al. 2004).

### Biofilm formation

**Fig. 5:**
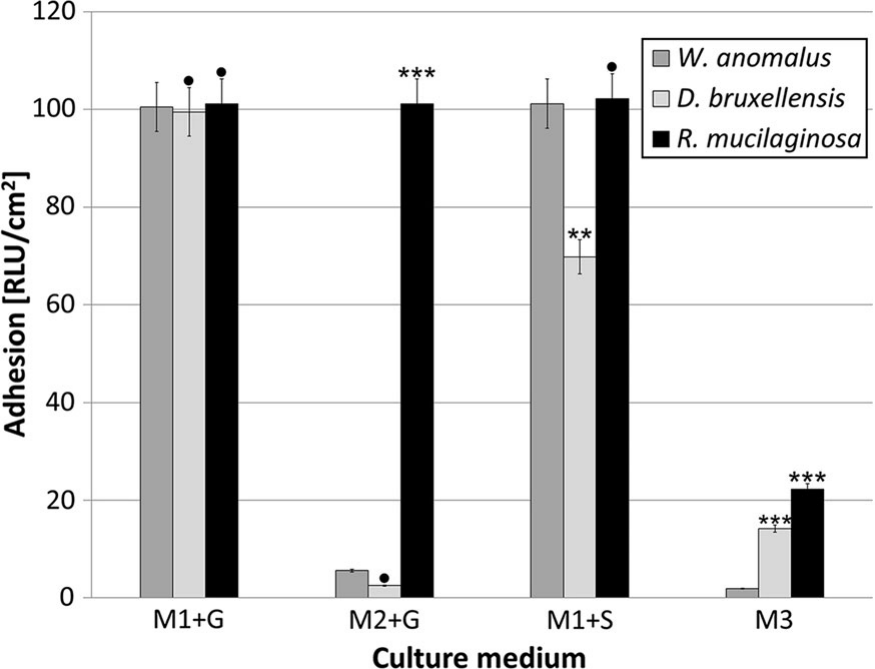
Adhesion of yeast isolates on glass surface after 6-day incubation. Values are means of three determinations ± standard deviation. Statistical differences between the obtained adhesion results were compared using a one-way repeated measures analysis of variance (ANOVA). Statistical significance was set at the level of 5% (p < 0.05). Values with the different markers are statistically different ^•^p ≥ 0.05; *p < 0.05; **p < 0.01; ***p < 0.001 (data were compared to those received for *W. anomalus*)

Coaggregation may also promote the development of biofilms (Vornhagen et al. 2013). Currently, short-term biofilm studies are usually performed in 96-well plates. The formation of biofilms in the well plates is thus limited to young biofilms (24–48 h old), which are not representative of the older or mature consortia found typically in industrial plants (O’Toole 2011). We used a 6-day incubation period for cultivation of yeast strains in culture media with glass carriers. The vertical position of the carriers in the test samples enabled observation of microbial adhesion on the carrier, at different heights in the culture medium. Previous studies had shown irregular cell adhesion may often be observed on the tested surface, and the best adhesion occurs at the interfaces between the carrier, air and the culture liquid medium (Kregiel 2013b; Kregiel et al. 2014). Luminometry (an analytical method based on ATP measurements) was used to determine the level of bacterial adhesion. This method has been shown to be effective at determining the quantity not only of microbial cells but also of organic substances on tested surfaces (Kregiel 2013a, b). The results of luminometric analysis, expressed as RLU/cm^2^, are presented in Fig. 5.

**Fig. 6:**
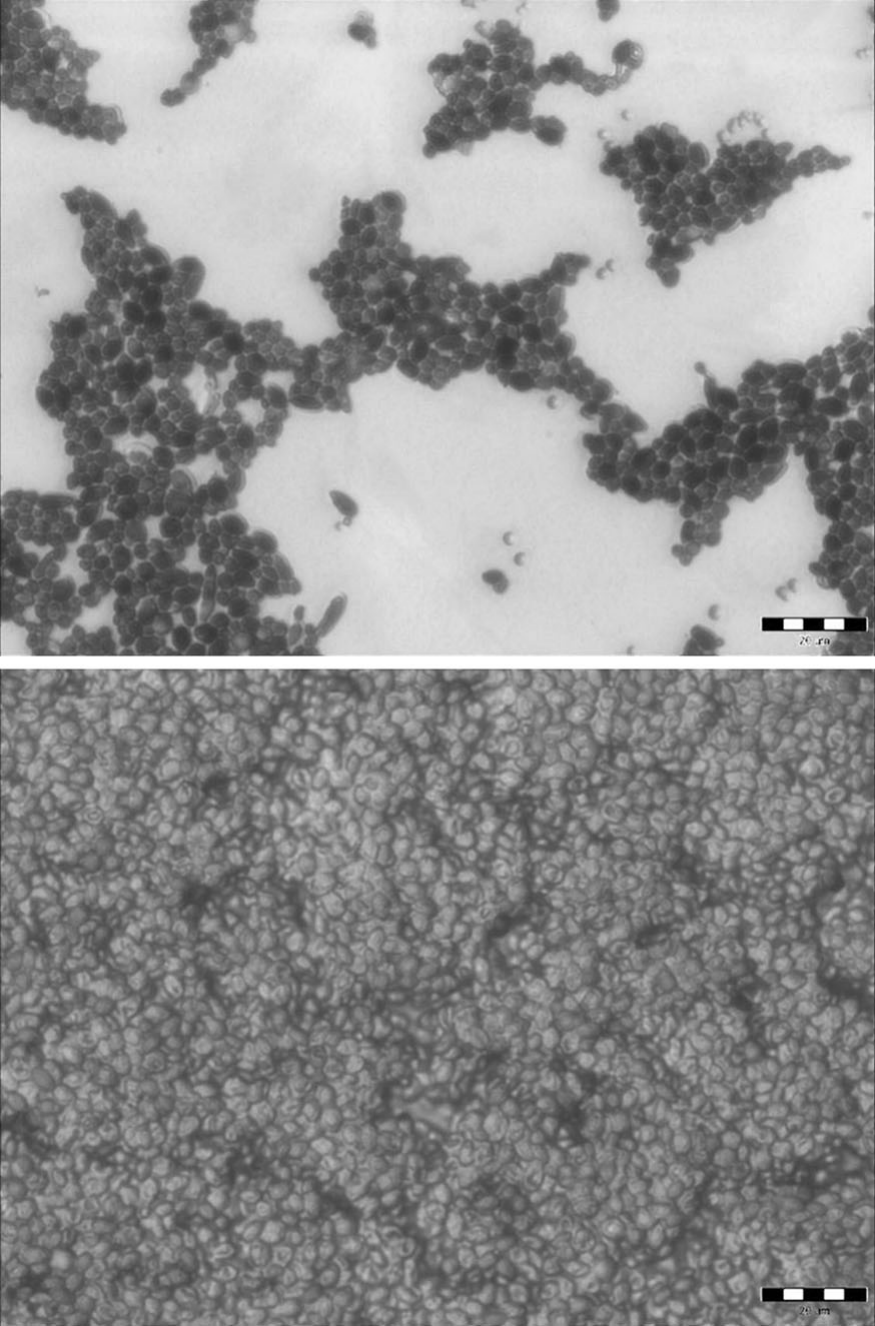
Attachment of *W. anomalus* cells (top) and *R. mucilaginosa* cells (bottom) to glass surface in flavored mineral water (M3 medium). Bar represents 20 μm

Yeast adhesion is an unusually complex process, in which various factors and mechanisms may be involved (Verstrepen and Klis 2006). In our study, biofilm formation was found to be genera- and culture medium-dependent, and yeast hydrophobicity was not always correlated with cell adhesion. After a 6-day incubation, the levels of adhesion in minimal medium M1 were very high (99–101 RLU/cm^2^), but biofouling was greatest for *R. mucilaginosa*, regardless of the type of culture medium used. Figure 6 shows images of glass surfaces stained with fuchsin. Yeast cell adhesion was detected on the glass material, with surface coverage ranging from around 20–100% of the total area.

Our results confirm the strong adhesive properties of *Rhodotorula* spp. cells reported by Gonzalez-Garcia et al. (2013). *W. anomalus* and *D. bruxellensis* also showed quite good adhesion abilities, especially in minimal culture medium M1. According to the literature, adhesion is activated mainly in minimal media by carbon and/or nitrogen starvation. In our study, the results were comparable for cell adhesion in minimal medium M1 with glucose or with sucrose. This fact suggests that the process of adhesion was sugar-insensitive. Sugar-independent adhesion is mediated by adhesins, which promote hydrophobic interactions between the cells and certain abiotic surfaces (Verstrepen and Klis 2006).

According to the literature, *W. anomalus* exhibits high agglutinating and adherence capacity (Jimoh et al. 2012). *D. bruxellensis* has been found on the surfaces of oak barrels and is known to produce biofilms that are difficult to remove (Joseph et al. 2007). Moreover, *D. bruxellensis* easily adapts to harsh and limiting environmental conditions, such as low pH, wide temperature ranges and ‘poor’ nitrogen sources (Schifferdecker et al. 2014). Its high tolerance to both ethanol and acetic acid, together with its ability to grow without oxygen, allow this yeast to cohabitate with other microorganisms in several different ecological niches (Moktaduzzaman et al. 2016). Flocculation or biofilm formation may help to protect the yeast cells in the middle of the flocs from the environment, away from stressors (Verstrepen and Klis 2006).

In the present study, the lowest levels of yeast adhesion were observed in commercial fruit-flavored mineral water (M3). However, in our previous investigations, the bacteria *Asaia* ssp. had shown good adhesion abilities in this medium, producing soluble or insoluble extracellular polymeric substances (Kregiel 2013a). On this basis, it can be assumed that *Asaia* spp. cells were responsible for the formation of flocs, but that, due to their hydrophobicity and production of slime, the presence of yeast cells in soft drinks helps to strengthen the overall structure of the flocs.

## Conclusion

In this study, cell co-aggregates in defective/spoiled soft drinks samples were identified as comprising bacterial *Asaia* spp. and yeasts belonging to three separate species, namely *W. anomalus*, *D. bruxellensis* and *R. mucilaginosa*. Significantly, the low pH of the beverages and the presence of chemical preservatives did not prevent the growth of spoilage microorganisms. Yeasts and bacteria formed stable flocs, and the microbiota was difficult to separate from each other. Extensive studies have been conducted into bacterial biofilms and cell aggregation. However, the co-existence of spoilage yeasts and bacteria is an under-researched area. Our results focus on the problems raised by mixed cultures formed by the relatively newly-identified acetic acid bacteria *Asaia* spp. Despite the relatively short period of our research and the limited possibilities for the detection of microbial contamination in commercial soft drinks, we have increased knowledge and understanding of the effects of these spoilage microorganisms in the soft drinks industry. This is important at the practical level of quality control and assurance, as well as providing impetus for further research into microbiota formed by *Asaia* spp. and yeasts.

## References

[CR1] Aneja KR, Dhiman R, Aggarwal NK, Kumar V, Kaur M (2014). Microbes associated with freshly prepared juices of citrus and carrots. Int J Food Sci.

[CR2] Antolak H, Czyzowska A, Kregiel D (2016). Black currant (*Ribes nigrum* L.) and bilberry (*Vaccinium myrtillus* L.) fruit juices inhibit adhesion of *Asaia* spp. Biomed Res.

[CR3] Barata A, Malfeito-Ferreiram M, Loureiro V (2014). The microbial ecology of wine grape berries. Int J Food Microbiol.

[CR4] Borghi E, Sciota R, Biassoni C, Morace G (2011). Cell surface hydrophobicity: a predictor of biofilm production in *Candida* isolates?. J Med Microbiol.

[CR5] Cappelli A, Ulissi U, Valzano M, Damiani C, Epis S, Gabrielli MG, Conti S, Polonelli L, Bandi C, Favia G, Ricci I (2014). A *Wickerhamomyces anomalus* killer strain in the malaria vector *Anopheles stephensi*. PLoS ONE.

[CR6] Coda R, Cassone A, Rizzello CG, Nionelli L, Cardinali G, Gobbetti M (2011). Antifungal activity of *Wickerhamomyces anomalus* and *Lactobacillus plantarum* during sourdough fermentation: identification of novel compounds and long-term effect during storage of wheat bread. Appl Environ Microbiol.

[CR7] Crotti E, Damiani C, Pajoro M, Gonella E, Rizzi A, Ricci I, Negri I, Scuppa P, Rossi P, Ballarini P, Raddadi N, Marzorati M, Sacchi L, Clementi E, Genchi M, Mandrioli M, Bandi C, Favia G, Alma A (2009). *Asaia*, a versatile acetic acid bacterial symbiont, capable of cross-colonizing insects of phylogenetically distant genera and orders. Environ Microbiol.

[CR8] Dengis PB, Ne´lissen LR, Rouxhet PG (1995). Mechanisms of yeast flocculation: comparison of top and bottom-fermenting strains. Appl Environ Microbiol.

[CR9] Farkas Z, Márki-Zay J, Kucsera J, Vágvölgyi C, Golubev WI, Pfeiffer I (2012). Characterization of two different toxins of *Wickerhamomyces anomalus* (*Pichia anomala*) VKM Y-159. Acta Biol Hung.

[CR10] Favia G, Ricci I, Marzorati M, Negri I, Alma A, Sacchi L, Bandi C, Daffonchio D (2008). Bacteria of the genus *Asaia*: a potential paratransgenic weapon against malaria. Adv Exp Med Biol.

[CR11] Frey-Klett P, Burlinson P, Deveau A, Barret M, Tarkka M, Sarniguet A (2011). Bacterial-fungal interactions: hyphens between agricultural, clinical, environmental, and food microbiologists. Microbiol Rev.

[CR12] Gamero A, Ferreira V, Pretorius IS, Querol A, Piskur J, Compagno C (2014). Wine, beer and cider: unravelling the aroma profile. Molecular mechanisms in yeast carbon metabolism.

[CR13] Gänzlea M, Ripari V (2016). Composition and function of sourdough microbiota: from ecological theory to bread quality. Int J Food Microbiol.

[CR14] Gattlen J, Zinn M, Guimond S, Körner E, Amberg C, Mauclaire L (2011). Biofilm formation by the yeast *Rhodotorula mucilaginosa*: process, repeatability and cell attachment in a continuous biofilm reactor. Biofouling.

[CR15] Gonzalez-Garcia Y, Hernandez R, Zhang G, Escalante FME, Holmes W, French WT (2013). Lipids accumulation in *Rhodotorula glutinis* and *Cryptococcus curvatus* growing on distillery wastewater as culture medium. Environ Prog Sustain Energy.

[CR16] Horsáková I, Voldřich M, Čeřovskỳ M, Sedláčková P, Šicenrová P, Ulbrich P (2009). *Asaia* sp. as a bacterium decaying the packaged still fruit beverages. Czech J Food Sci.

[CR17] James SA, Collins MD, Roberts IN (1994). Genetic interrelationship among species of the genus *Zygosaccharomyces* as revealed by small-subunit rRNA gene sequences. Yeast.

[CR18] Jimoh SO, Ado SA, Ameh JB, Whong CMZ (2012). Characteristics and diversity of yeast in locally fermented beverages sold in Nigeria. World J Eng Pure Appl Sci.

[CR19] Joseph CML, Kumar G, Su E, Bisson LF (2007). Adhesion and biofilm production by wine isolates of *Brettanomyces bruxellensis*. Am J Enol Vitic.

[CR20] Katsikogianni M, Missirlis YF (2004). Concise review of mechanisms of bacterial adhesion to biomaterials and of techniques used in estimating bacteria-material interactions. Eur Cell Mater.

[CR21] Kregiel D (2013). Attachment of *Asaia lannensis* to materials commonly used in beverage industry. Food Control.

[CR22] Kregiel D (2013). Adhesion of *Aeromonas hydrophila* to glass surfaces modified with organosilanes. Food Technol Biotechnol.

[CR23] Kregiel D (2015). Health safety of soft drinks: contents, containers, and microorganisms. Biomed Res Int.

[CR24] Kregiel D, Rygala A, Libudzisz Z, Walczak P, Oltuszak-Walczak E (2012). *Asaia lannensis*—the spoilage acetic acid bacteria isolated from strawberry-flavored bottled water in Poland. Food Control.

[CR25] Kregiel D, Otlewska A, Antolak H (2014). Attachment of *Asaia bogorensis* originating in fruit-flavored water to packaging materials. Biomed Res Int.

[CR26] Kreth J, Herzberg MC, de Paz Chávez LE, Sedgley CM, Kishen A (2015). Molecular principles of adhesion and biofilm. The root canal biofilm.

[CR27] Marsh AJ, O’Sullivan O, Hill C, Ross RP, Cotter PD (2014). Sequence-based analysis of the bacterial and fungal compositions of multiple kombucha (tea fungus) samples. Food Microbiol.

[CR28] Mazzobre MF, Román MV, Mourelle AF, Corti HR (2005). Octanol–water partition coefficient of glucose, sucrose, and trehalose. Carbohydr Res.

[CR29] Moktaduzzaman M, Galafassi S, Vigentini I, Foschino R, Corte L, Cardinali G, Piškur J, Compagno C (2016). Strain-dependent tolerance to acetic acid in *Dekkera bruxellensis*. Ann Microbiol.

[CR30] Moore JE, McCalmont M, Xu J, Millar BC, Heaney N (2002). *Asaia* sp., an unusual spoilage organism of fruit-flavored bottled water. Appl Environ Microbiol.

[CR31] Nunes JM, Bizerra FC, Ferreira RC, Colombo AL (2013). Molecular identification, antifungal susceptibility profile, and biofilm formation of clinical and environmental *Rhodotorula* species isolates. Antimicrob Agents Chemother.

[CR32] O’Donnell K, Reynolds DR, Taylor JW (1993). *Fusarium* and its near relatives. The fungal holomorph: mitotic, meiotic and pleomorphic speciation in fungal systematic.

[CR33] O’Toole GA (2011). Microtiter dish biofilm formation assay. J Vis Exp.

[CR34] Sampaio JP, Gadanho M, Santos S, Duarte FL, Pais C, Fonseca A, Fell JW (2001). Polyphasic taxonomy of the basidiomycetous yeast genus *Rhodosporidium*: *Rhodosporidium kratochvilovae* and related anamorphic species. Int J Syst Evol Microbiol.

[CR35] Schifferdecker AJ, Dashko S, Ishchuk OP, Piškur J (2014). The wine and beer yeast *Dekkera bruxellensis*. Yeast.

[CR36] Sedláčková P, Čeřovskỳ M, Horsáková I, Voldřich M (2011). Cell surface characteristic of *Asaia bogorensis*—spoilage microorganism of bottled water. Czech J Food Sci.

[CR37] Teoh AL, Heard G, Cox J (2004). Yeast ecology of Kombucha fermentation. Int J Food Microbiol.

[CR38] Verstrepen KL, Klis FM (2006). Flocculation, adhesion and biofilm formation in yeasts. Mol Microbiol.

[CR39] Vornhagen J, Stevens M, McCormick D, Dowd SE, Eisenberg JNS, Boles BR, Rickard AH (2013). Coaggregation occurs amongst bacteria within and between domestic showerhead biofilms. Biofouling.

[CR40] Wirth F, Goldani LZ (2012). Epidemiology of *Rhodotorula*: an emerging pathogen. Interdiscip Perspect Infect Dis.

